# Improving Practice and Performance in Basketball

**DOI:** 10.3390/sports7090197

**Published:** 2019-08-27

**Authors:** Aaron T. Scanlan, Vincent J. Dalbo

**Affiliations:** Human Exercise and Training Laboratory, School of Health, Medical and Applied Sciences, Central Queensland University, Rockhampton 4702, Australia

Basketball is ranked in the top three team sports for participation in the Americas, Australia, Europe, Southeast Asia, and Western Pacific nations, making it one of the most popular team sports worldwide [1]. The physical demands and high popularity of basketball present a wide range of potential applications in society. At one end, basketball may offer a vehicle to combat high inactivity rates and reduce economic health burdens for government officials and health administrators in many countries due to the popularity of the game combined with the evidence supporting recreational basketball eliciting intense physical demands with low perceptual demand [2]. At the other end, professional basketball competitions have emerged in over 100 countries with more than 70,000 professional players globally [3], creating a lucrative business that provides legitimate career pathways for players and entertainment for billions of people. Despite the wide range in application, it is surprising how little research has been conducted in basketball relative to other sports. For instance, a rudimentary search on PubMed showed basketball to yield considerably less returns than other sports with a similar global reach and comparable returns to sports governed in less regions of the world (Table 1). Consequently, we sought to edit a Special Issue on “Improving Practice and Performance in Basketball” to provide a collection of studies from basketball researchers across the world and increase available evidence on pertinent topics in the sport. In total, 40 researchers from 16 institutions or professional bodies across nine countries contributed 10 studies in the Special Issue.

Most research conducted in basketball has focused on athletic populations. For instance, a review of the 228 studies returned on PubMed for “basketball” in 2019 (up to August 9th) indicates over 25% of studies focused on the incidence, treatment, rehabilitation, or screening of injuries, while 11% of studies described physical, fitness, or functional attributes in competitive basketball players. These trends emphasize the strong interest in understanding injury prevention and treatment in basketball, as well as attributes which may underpin successful players, both of which are oriented towards optimizing player and team performance. Regarding enhancing performance, an increasingly popular field of research in basketball is examining monitoring methods (7% of PubMed studies in 2019) to better understand demands placed on players across the season and provide evidence for decision-making regarding player management. Several reviews have recently been published highlighting the interest in quantifying game [4] and training demands [5], using heart rate monitoring [6], and applying microsensors to measure player workloads [7] in competitive basketball. Available monitoring technologies provide basketball coaches and high-performance staff with a plethora of data regarding player fitness, workloads, and fatigue status to inform decisions regarding training prescription and recovery opportunities for minimizing injury risk and optimizing performance. In turn, basketball research has readily used game-related statistics (3% of PubMed studies in 2019) to describe player and team performance, which provide an expansive reservoir of data, usually publicly available, to link outcomes of interest to performance. Consequently, our Special Issue was open to research exploring various current topics that have potential to impact practice in basketball. 

In keeping with the recent trends in basketball research, the Special Issue contains two reviews with one focused on exploring the utility of various monitoring strategies to detect player fatigue [8] and the other identifying issues to consider around the extensive travelling requirements in the National Basketball Association (NBA), the premier global basketball competition [9]. Both reviews highlight the practical aspects relating fatigue and travel in basketball, including potential implications for injury, workload management, recovery, and assessment in players. Furthermore, two applied studies in the Special Issue examine workload monitoring in basketball, with one exploring the impact of game scheduling on accelerometer-derived workload [10] and the other examining changes in jump kinetics and perceptual workload across the season [11]. An additional three studies in the Special Issue identified game-related statistics explaining game outcomes and regional differences in various elite competitions (Olympics [12], EuroBasket [13], and Continental Championships [14]). The remaining three studies described physical [15,16] and skill [17] attributes in various player samples. It should also be noted our Special Issue addresses an important issue of increasing research in female athletes, who have traditionally been under-represented in the literature compared to male basketball players, with seven of the eight original studies (88%) containing female basketball players. 

The immediate future of basketball research in high-performance settings is highlighted by issues faced in practice. Specifically, key players are missing games or being rested for “load management” in the NBA to reduce player injury risk, despite some initial evidence suggesting greater rest during the regular season (6 ± 1 vs 1 ± 1 games) does not reduce injury incidence or performance in the playoffs [18]. Likewise, condensed game schedules [19] and the total minutes played in individual games [20] have been shown to have no significant effects on injury risk in NBA players. In contrast, other research suggests the total number of games played in a season impacts injury risk in the NBA [21], highlighting the need for further research on this topic to gather a definitive understanding regarding the effects of managing player workloads on injury risk. In fact, more research needs to build upon the extensive descriptive evidence already available and identify modifiable factors contributing to injuries in basketball players for coaches and high-performance staff to control risk as much as possible. In addition to injury, future basketball research should seek to further examine the efficacy of logical and practical intervention strategies on player performance. For example, an increasing number of studies are examining the utility of different training approaches, including resistance training [22], court-based conditioning [23], and games-based drills [5], as well as nutritional strategies [24,25,26,27] and recovery practices [28] on performance outcomes. Furthermore, it is integral for future research assessing player performance to use basketball-specific assessments. In this regard, more research is recognizing the need for greater specificity in measuring performance in basketball, with an increased number of studies exploring the utility of basketball-specific testing protocols to assess relevant physical attributes [29,30,31,32] as well as in-game statistics [33] and workloads [34] to quantify player performance in a robust manner with increased application to actual competition. 

## Figures and Tables

**Table 1:** Returns on Scopus for basketball relative to other sports.

Search Term (Sport)	Number of Returns	Number of Countries Played in *
Soccer	4176	211
Tennis	2188	211
Basketball	1427	213
Baseball OR Softball	1250	141
“American football” OR NFL	1150	104
Hockey	993	137 ^†^
Cricket	940	104
“Rugby union” OR “Rugby sevens” OR “Rugby league”	808	119 ^‡^

Note: Search conducted on August 9th 2019 via https://www.ncbi.nlm.nih.gov/pubmed/ and was restricted to past five years. * The number of countries identified as members by the international governing body; ^†^ field hockey included 137 member countries, while ice hockey included 76 member countries; ^‡^ rugby union included 119 member countries, while rugby league included 68 member countries.
